# Fatal Case of Chorea

**Published:** 1827-03

**Authors:** 

**Affiliations:** *the* Middlesex Hospital


### CHOREA.

### *Fatal Case of Chorea, treated at the* Middlesex Hospital, *by* Dr. Hawkins.

Elizabeth Smith, aged seventeen, was admitted into the hospital on the 5th September, 1826. It was stated by her friends, that seven weeks previously she had suffered from a severe attack of rheumatism, chiefly in her knees and shoulders. This had got better; but a fortnight ago she was seized with involuntary convulsive motions, affecting the muscles of the legs and arms, and neck. These had continued ever since, with extreme violence. The catamenial discharge had formerly made its appearance, but had been suppressed for four months. She complained of headache, thirst, and pain in her back. Pulse ninety-six; tongue foul; bowels costive.

A full but unsuccessful trial was in this case made of the purgative plan of Dr. Hamilton. The patient had repeated doses of Calomel, Senna, or Jalap; and an enema of Oil of Turpentine was three times administered. These measures never failed to bring away copious and dark-coloured evacuations, but they procured not the smallest alleviation of the convulsive spasms, which resembled those with which hydrophobia exhausts its victim. It seemed impossible for the patient to hold her head still for a single instant; and the grinding of the teeth was so violent as at last to force out the incisores from the lower jaw. The convulsions continued without any remission, except during some short intervals of broken and interrupted sleep. The patient's articulation was rendered difficult, but never entirely stopped; and her consciousness did not appear to be at any time impaired.

On the second night after her admission, she was ordered to be put into a warm bath, with the hope of bringing on the catamenial discharge; but this measure unfortunately aggravated the convulsions, and produced such an accession of irritation, and even of inflammatory symptoms, that it was deemed necessary to take from her sixteen ounces of blood. The blood drawn presented an inflammatory appearance. The bleeding was repeated on the following day, but was succeeded by very little alleviation of the spasms.

After the failure of purgative medicines, a short trial was given to Musk, but it produced no effect upon the symptoms. Some pills, consisting of three grains of Camphor and one of Opium, succeeded in procuring sleep. After the second administration of these remedies, she slept soundly; but upon waking she sunk gradually, and expired on the morning of the 13th September (the sixth day after her admission), apparently exhausted by the violence and long continuance of the spasms.

Upon examination after death, no morbid appearance was discovered within the cranium. The duplicatures of the pleura adhered firmly together. In the upper part of the lungs there were a few tubercles of a large size, and several earthy concretions were deposited in various parts. Adhesions were also formed between the external membrane of the liver and the surrounding portions of the peritoneum. The intestines were healthy in appearance. The omentum and mesentery were studded wlth numerous cysts; some containing a black semi-fluid matter, others containing calcareous depositions. Several large concretions of the same kind were found in the substance of the pancreas. The uterus was somewhat large and vascular, and the mucous lining of its body and fundus were highly injected. The neck of the uterus contained a little gelatinous matter; by which, however, it did not appear to be completely closed. The fallopian tubes and ovaries contained a good deal of the black matter which has been before described. There were also some small vesicles, of a whitish colour, within the ovaries; but there did not appear to be any distinct corpus luteum.

The foregoing case has been detailed for the sake of preserving an account of the examination after death. Chorea, having seldom occurred with such extreme violence, has seldom had a fatal termination. An examination, therefore, of morbid appearances after such an event must be interesting to the pathologist, on account of its rarity. There are no cases of this disease to be met with in MORGAGNI. LIEUTAUD has a section on the morbid appearances observed after fatal cases of convulsions; as likewise after some cases of hysteria and of chlorosis. But there are none of these which exactly correspond with the case which has been described.

It has generally been supposed that irritation of the brain or nervous system is the proximate cause of chorea. Sufficient cause for such irritation was met with in the preceding case. But a question might arise, whether it were chiefly caused by the numerous earthy concretions, which might have served to irritate the nerves of all the abdominal viscera; or whether from the highly excited state of the uterine system.

The German writers have drawn a distinction between what they have termed the German or great St. Vitus's dance, and the minor St. Vitus's dance, or chorea of the English. The foregoing case did not coincide in its symptoms with either the one or the other, as they have been described by these authors. The convulsions were far more violent than are common in the latter; and they wanted the regular paroxysms and intermissions which have been attributed to the former.

The foregoing case was said to be preceded by a severe attack of rheumatism. It is worthy of remark, that rheumatic pains are said to attend the commencement of each of the cases which Dr. Hamilton, in his observations on Chorea, has cited from Stoll's Rat. Medendi. These cases were severe, and, after the failure of stimulant and antispasmodic medicines, were ultimately cured by purgatives. It is a question whether the muscular pains complained of in such instances were really rheumatic; or whether they might not have arisen from nervous irritation, or from whatever else might have been the proximate cause of the subsequent spasms.

